# SMYD3: a new regulator of adipocyte precursor proliferation at the early steps of differentiation

**DOI:** 10.1038/s41366-023-01450-x

**Published:** 2023-12-26

**Authors:** Tatjana Sajic, Chayenne Karine Ferreira Gomes, Marie Gasser, Tiziana Caputo, Nasim Bararpour, Esther Landaluce-Iturriria, Marc Augsburger, Nadia Walter, Alexandre Hainard, Isabel C. Lopez-Mejia, Tony Fracasso, Aurélien Thomas, Federica Gilardi

**Affiliations:** 1 Unit of Forensic Toxicology and Chemistry, CURML, Lausanne and Geneva University Hospitals, Lausanne, Geneva Switzerland; 2 Faculty Unit of Toxicology, CURML, Faculty of Biology and Medicine, University of Lausanne, Lausanne, Switzerland; 3 Section on Integrative Physiology and Metabolism, Joslin Diabetes Center, Harvard Medical School, Boston, MA USA; 4 Stanford Center for Genomics and Personalized Medicine, Stanford, CA USA; 5 Department of Genetics, Stanford University, Stanford, CA USA; 6 Center for Integrative Genomics, University of Lausanne, Lausanne, Switzerland; 7 Proteomics Core Facility, Faculty of Medicine, University of Geneva, Geneva, Switzerland; 8 Unit of Forensic Medicine, CURML, Lausanne and Geneva University Hospitals, Lausanne, Geneva Switzerland

**Keywords:** Cell biology, Fat metabolism

## Abstract

**Background:**

In obesity, adipose tissue undergoes a remodeling process characterized by increased adipocyte size (hypertrophia) and number (hyperplasia). The ability to tip the balance toward the hyperplastic growth, with recruitment of new fat cells through adipogenesis, seems to be critical for a healthy adipose tissue expansion, as opposed to a hypertrophic growth that is accompanied by the development of inflammation and metabolic dysfunction. However, the molecular mechanisms underlying the fine-tuned regulation of adipose tissue expansion are far from being understood.

**Methods:**

We analyzed by mass spectrometry-based proteomics visceral white adipose tissue (vWAT) samples collected from C57BL6 mice fed with a HFD for 8 weeks. A subset of these mice, called low inflammation (Low-INFL), showed reduced adipose tissue inflammation, as opposed to those developing the expected inflammatory response (Hi-INFL). We identified the discriminants between Low-INFL and Hi-INFL vWAT samples and explored their function in Adipose-Derived human Mesenchymal Stem Cells (AD-hMSCs) differentiated to adipocytes.

**Results:**

vWAT proteomics allowed us to quantify 6051 proteins. Among the candidates that most differentiate Low-INFL from Hi-INFL vWAT, we found proteins involved in adipocyte function, including adiponectin and hormone sensitive lipase, suggesting that adipocyte differentiation is enhanced in Low-INFL, as compared to Hi-INFL. The chromatin modifier SET and MYND Domain Containing 3 (SMYD3), whose function in adipose tissue was so far unknown, was another top-scored hit. SMYD3 expression was significantly higher in Low-INFL vWAT, as confirmed by western blot analysis. Using AD-hMSCs in culture, we found that SMYD3 mRNA and protein levels decrease rapidly during the adipocyte differentiation. Moreover, SMYD3 knock-down before adipocyte differentiation resulted in reduced H3K4me3 and decreased cell proliferation, thus limiting the number of cells available for adipogenesis.

**Conclusions:**

Our study describes an important role of SMYD3 as a newly discovered regulator of adipocyte precursor proliferation during the early steps of adipogenesis.

## Introduction

The etiology of obesity is multifactorial and involves an interaction between genetic and environmental factors [1]. Obesity is driven by the unbalance between calorie intake and consumption, which results in an abnormal accumulation of white adipose tissue (WAT). WAT expansion occurs through both hyperplasia, by favoring the differentiation of adipocyte precursors to increase the number of adipocytes, and hypertrophy, by enlarging the size of existing adipocytes [2]. In addition, vWAT undergoes massive remodeling including changes in tissue cellular composition, with recruitment of pro-inflammatory immune cells. The activation of this local inflammatory response is considered as a key event in the development of the detrimental consequences of obesity, such as metabolic syndrome [3]. Interestingly, however, many individuals with obesity are relatively resistant to developing these complications [4–6], which raises questions about possible factors modulating the susceptibility to obesity-driven inflammation and its deleterious metabolic consequences [7].

In the last years, converging reports have suggested that the ability to recruit new fat cells through adipogenesis, which would favor the hyperplastic over the hypertrophic expansion of the tissue, is a critical determinant of a healthy adipose tissue remodeling with reduced activation of pro-inflammatory pathways in obesity [7]. During adipogenesis, mesenchymal precursors first commit themselves to the adipocyte lineage. This step is followed by terminal differentiation, where committed pre-adipocytes acquire the characteristics of mature adipocytes. The regulation of this differentiation process has been extensively studied over the past three decades using, in particular, several fibroblast-like cell culture models that differentiate to adipocytes in response to a hormonal cocktail (reviewed in [8]). However, which molecular players would favor adipogenesis to drive a healthy tissue expansion in vivo, and how, is far from being understood. Among others, epigenetic mechanisms, which can alter gene expression in response to environmental inputs, seem very good candidates as fine-tuning regulators of the individual vulnerability to obesity-driven detrimental consequences.

Epigenetics refers to chemical modifications, including acetylation, methylation, phosphorylation, ubiquitination etc. of either single nucleotides and/or histones that occur without a change in the DNA sequence. These changes can profoundly affect gene transcription as well as DNA replication [9]. Several groups reported effects of epigenetic regulators on adipogenesis. For example, (1) class I Histone deacetylases (HDACs), particularly HDAC3, have emerged as important regulators of adipocyte differentiation which drives these cells toward a brown phenotype [10–12]. (2) The histone methyltransferase G9a promotes the di-methylation of the histone H3K9 in the promoter of PPARγ gene, thereby blocking its transcription and subsequently adipocyte differentiation [13]. (3) The histone methyltransferase SETDB1 mediates H3K9 trimethylation on PPARγ and CEBPα genes, thus keeping their expression low and allowing adipocytes to remain primed for differentiation [14], (4) while the histone lysine demethylase 1 (LSD1) promotes adipocyte differentiation by decreasing H3K9 dimethylation at the CEBPα promoter [15].

Here, we took advantage of a sub-set of visceral white adipose tissue (vWAT) samples collected from C57BL6 mice fed with a HFD for 8 weeks showing a low susceptibility to the onset of adipose tissue inflammation, that we called low inflammation (Low-INFL), as opposed to their high inflammation (Hi-INFL) counterpart. We applied Data-Independent Acquisition Mass Spectrometry (DIA-MS) based proteomic analysis on vWAT samples from Low-INFL and Hi-INFL mice and quantified more than 6000 proteins. We experimentally validated our results by orthogonal analytical approaches and functional in-vitro experiments that allowed us to identify the chromatin modifier SET And MYND Domain Containing 3 (SMYD3) as a new regulator of adipocyte precursor proliferation at the early steps of adipogenesis.

## Material and methods

### Animal experiments

All animal experiments were approved by the Swiss Veterinary Office (VD-2942.b) and were previously described [16]. In brief, C57/BL6 male mice were purchased from Janvier Labs and housed 5 per cage. Four-week-old mice were fed for 2 weeks with a 10% calories from fat control diet (D12450J, Research Diet). At 6 weeks mice were either shifted to a high-fat diet (HFD) containing 60% calories from fat (D12492, Research Diet) or kept on a control diet for 8 weeks (*n* = 60 for control and *n* = 53 for HFD). Random blocking was used. All animals were kept in a 12:12 h light:dark cycle with water and food ad libitum. All the mice were sacrificed using CO2 between ZT2 and ZT5, where ZT0 corresponds to the light onset time.

The onset of visceral adipose tissue inflammation after 8 weeks of HFD was assessed by measurement of the following parameters: circulating levels of insulin, resistin and leptin levels, and expression of *Cxcl212*, *Ccl2* and *Itgax* in vWAT in all HFD mice as compared to 20 randomly picked control mice. All these measurements, in addition to the individual mouse weight, were used as variables to perform a Principal Component Analysis (PCA) and HFD-fed mice were classified as Low Inflammation (Low-INFL) when they were clustering close to the control group, as opposed to the High Inflammation (Hi-INFL) mice [16].

### Plasma biochemistry

Circulating levels of insulin, resistin and leptin were simultaneously measured in plasma samples using a ProcartaPlex Multiplex Immunoassay (Life Technologies Europe, Switzerland), on a Luminex 200 system, according to the manufacturers’ instructions.

### Proteomics analysis of vWAT by data-independent acquisition mass spectrometry (DIA-MS)

Proteomic analysis was performed starting from 30 mg of snap-frozen visceral adipose tissue per mice (*n* = 6). Samples were homogenized in 500 µl of ice-cold phosphate-buffered saline (PBS, #10010015, Gibco) and the soluble tissue proteins were precipitated with trichloroacetic acid (TCA) and washed with ice-cold acetone.

Purified protein pellets were dissolved in 8 M urea buffer and digested overnight with a ratio of 1 μg trypsin (#V5113, Promega) for 20 μg protein. Generated peptide digests were cleaned on MACROSpin Plate-Vydac Silica C18 (Nest Group Inc. Southborough, MA), solubilized in 30 μL of 0.1% aqueous formic acid (FA) with 2% acetonitrile (ACN). Indexed retention time (iRT) peptides were added (RT-kit WR, Biognosys) in equal 1 pmol/μL amount into each sample prior to mass spectrometry (MS) injection. Peptides digests of respective samples were processed by liquid chromatography-electrospray ionization tandem mass spectrometry (LC-ESI-MS/MS) on an Orbitrap Fusion Lumos Tribrid mass spectrometer (Thermo Fisher Scientific) equipped with an Easy nLC1200 liquid chromatography system (Thermo Fisher Scientific). Raw data analysis, generation of peptide and protein matrices were performed with commercial proteomic software Spectronaut (version:14.8.201029.47784, Biognosys, https://biognosys.com/software/spectronaut/) as described previously [17, 18]. The successive steps of LC-MS analysis and raw data processing are detailed in Supplementary material.

### Statistical analysis and visualization of vWAT proteomics data

R software for statistical computing and graphics (version:3.6.1) was used for data analysis and visualization. To explore the changes induced by HFD in Low-INFL and Hi-INFL groups, we performed Limma analysis [19] on generated protein matrix. Two-sided *p*-values were adjusted for the number of tests performed via a Benjamini–Hochberg (BH) FDR-based correction (adj.p) and proteins with adj.*p*< 0.05 and fold change (FC) ≥ 1.5 were considered as differentially expressed.

To independently select the most descriptive features for each tissue group from large protein data matrix (6051 protein), we used Supervised Partial Least Squares Discriminant Analysis (PLS-DA) through mixOmics’ R package (version 6.10.9) and imputed 3 components with limited number of features per component (*N* = 100).

GO Enrichment analysis was performed using R package Disease Ontology (DO) Semantic and Enrichment (DOSE, version 3.14.3) [20]. As input lists, we used differential proteins from each respective comparison. UniProt IDs were converted to GeneIDs and enrichment analysis was performed for biological process or “BP” subontology against mouse genome database “org.Mm.eg.db.” and under FDR control set up to 0.05. We reported all enrichment GO categories with FDR < 0.05 and with minimum 3 and maximum 50 genes annotated by Ontology term.

### Cell culture and treatment

Adipose-Derived human Mesenchymal Stem Cells (AD-hMSCs, Lifeline Cell Technology, Frederick, MD, USA) were expanded at 37°C and 5% CO2 in Mesenchimal Stem Cell Growth Medium 2, supplemented with Mesenchymal Stem Cell Growth Medium 2 Supplement Mix (PromoCell, Heidelberg, Germany). For differentiation experiments, confluent cells were switched to Mesenchymal Stem Cell Growth Medium 2 supplemented with 10% Foetal Bovine Serum (Biowest #S1810-500, Nuaillé, France), 0.2 μΜ Indomethacine (Sigma-Aldrich, #I7378), 10 μg/ml insulin (Sigma-Aldrich #I2643), 1 μM dexamethasone (Sigma-Aldrich #D2915), and 0.5 mM Isobutylmethylxanthine (Sigma-Aldrich, #I7018), to induce the differentiation into adipocytes. Medium was replenished every three days. For cell growth measurements, cells were detached with TrypLE Express (Gibco, #12304-021, Thermofisher) and were counted with a Cell Countess II FL (Thermo-Fisher Scientific).

### RNA silencing

AD-hMSCs at 70% of confluence were detached with TrypLE (Gibco), transfected with 20 nM of hSMYD3 Silencer Select pre-designed siRNAs (Ambion, clone s34865, Thermo-Fisher) or Silencer™ Negative Control (Ambion #AM4611) using Lipofectamine RNAiMAX Reagent (Invitrogen, #13778. Thermo-Fisher), following manufacturer’s instructions, and plated. After 48 h, undifferentiated cells were harvested, or differentiation was induced as described above.

### Proliferation assay

Cell proliferation was assessed by using the Click-iT EdU Alexa Fluor 488 Flow Cytometry Assay Kit (Thermo-Fisher Scientific #C10425). 48 h after SMYD3 gene silencing, 10 μM 5-Ethynyl-2′-deoxyuridine (EdU) was added to the cells for 4 h in concomitance with the adipogenic cocktail, when indicated. Cells were collected, fixed and stained according to manufacturer’s instructions. DNA was stained with FxCycle Violet Ready Flow Reagent (Thermo-Fisher Scientific #R37166) and the percentage of cells in S phase was determined with a CytoFlex flow cytometer (Beckman-Colter) using the CytExpert software, version 2.4.

In addition, we used the Cell Proliferation Kit I (MTT) (Roche Diagnostics, #11465007001, Mannheim, Germany) following manufacturer instructions. The plates were incubated at 37°C and 5% CO_2_ for 20 min with the MTT reaction mix and DMSO was used to extract the coloration. The absorbance was measured in duplicate at 540 nm with an Infinite M Nano Reader (Tecan, Männedorf, Switzerland).

### Lipid staining

After 14 days of differentiation either Oil Red O or Nile Red were used for lipid staining. For Oil Red O, cells were fixed with formaldehyde for 15 min. The staining solution (Sigma-Aldrich #O1391) was diluted 60:40 in distilled water, filtered after 1 h and added to dishes for 4 h. Excessive staining solution was removed and cells were washed twice with distilled water. After taking pictures, the lipid staining was extracted from the whole well with DMSO and the absorbance was measured in duplicate at 455 nm with an Infinite M Nano Reader (Tecan, Männedorf, Switzerland). For Nile Red staining, AD-hMSCs were transfected onto 96 well plates and differentiated. At day 14 of differentiation, cells were washed twice with PBS before a 15 min fixation in 4% formaldehyde at RT. The fixed cells were washed twice with PBS before a 15 min staining with Nile Red 5 µg/mL to stain cellular lipids. Nuclei were counterstained with 2 μg/ml of Hoechst, and the cells were washed again before imaging. Images (4 fields per well, 4 wells per condition) were acquired for each independent biological replicate using a Celena X High content imaging system (Logos Biosystems). The lipid area was quantified using the proprietary Cell analyzer software (Logos Biosystems) and normalized by nuclei count (used as a proxy for cell number).

### RNA extraction and quantitative PCR

Total RNA was isolated from undifferentiated and differentiated cells, or from adipose tissue, using the Direct-zol RNA MiniPrep Kit (ZymoResearch, #R2052, Lucerna-Chem, Luzern, Switzerland) following manufacturer protocol. cDNA was synthesized using 100 ng of total RNA with the iScript cDNA Synthesis Kit (BioRad, #1708891, Cressier, Switzerland) following manufacturer instructions. For real-time quantitative PCR, KAPA PROBE FAST qPCR Master Mix (2X) Kit or KAPA SYBR FAST qPCR Master Mix (2X) Kit were used (KapaBiosystems, #KK4703 or #KK4602, Sigma-Aldrich). The primer sets are shown in Supplementary Table 1. *36b4* or RPS13 were used as a housekeeping genes in mouse and human samples, respectively, and the relative expression was calculated with the 2^–∆∆Ct^ method.

### Western blotting

Whole proteins were extracted using mPER Mammalian Protein Extraction Reagent (Thermofisher, #78501) supplemented with Halt protease inhibitor (Thermofisher, #78426) and Halt phosphatase inhibitor (Thermofisher, #78429) cocktails. For adipose tissue extracts, the lysates were left 1 h at 4 °C on a rotating wheel and then sonicated 5 cycles 30″ ON/30″ OFF, using a Bioruptor Pico (Diagenode, Liège, Belgium). Histone extracts were isolated from 1 × 10^6^ undifferentiated and differentiated cells using the histone extraction kit (Abcam #AB113476). Protein concentration was determined by Pierce BSA protein assay Kit (Pierce, #23227, Thermofisher). Identical amounts of lysates (10–15 μg for total, 21 μg for histone extracts) were resolved by SDS-PAGE. Anti-SMYD3 (Diagenode, #C15410253, used at 1:1000), anti-GAPDH (Cell signaling, #2118s, used 1:1000), anti H3K4me3 (Diagenode #C15410030, used at 1:1000), anti-rabbit HRP for ECL (GE Healthcare, #NA934V, used at 1:10000) antibodies were used for western blot. Detection was performed with ECL Select kit (Cytiva, #RPN2235, Amersham,) in a Syngene G:BOX. Quantification of band density was performed with ImageJ.

### Statistical analysis

For proteomics studies, statistical analyses were performed in the R environment, as described above. For cell experiments, experiments were performed at least in triplicate and repeated at least three times. Data are represented as mean± SEM of the independent experiments, unless differently indicated in the legend. Statistical tests were performed using GraphPad Prism version 9.1.0 for Windows (GraphPad Software, San Diego, CA, USA, www.graphpad.com).

## Results

**Fig. 1:**
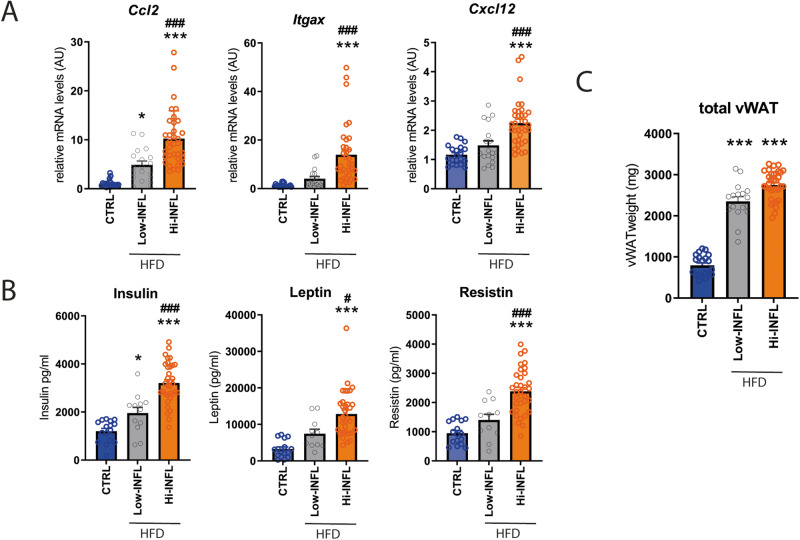
Limited inflammatory response to HFD in low inflammation (Low-INFL) mice. C57/BL6 male mice were fed for 8 weeks with control or HFD diet. Based on the onset of vWAT inflammation the mice fed with HFD were further divided in two groups. Low Inflammation (Low-INFL) mice had lower mRNA levels of markers of inflammatory cell infiltration (i.e. *chemokine (C-C motif) ligand 2 (Ccl2)*, Integrin Subunit Alpha X (*Itgax)*, and C-X-C motif chemokine ligand 12 (*Cxcl12*)) compared to high inflammation (Hi-INFL) mice **A** and lower levels of circulating insulin, leptin and resistin **B**. The total mass of vWAT was similarly increased in Low-INFL and Hi-INFL mice **C**. *n* = 20 for control diet; *n* = 19 for Low-INFL; *n* = 34 for Hi-INFL. Bars represent mean ± SE. ^*^P < 0.05, ^***^P < 0.001 versus control group; ^#^*P* < 0.05, ^###^*P* < 0.001 vs. Low-INFL group, as calculated by one-way ANOVA followed by Tukey’s multiple comparisons test.

To identify new players in the fine-tuning of WAT expansion in response to nutritional challenges (i.e. HFD), we investigated a set of vWAT samples collected from C57/BL6j male mice fed either a HFD or a control diet for 8 weeks [16]. As expected, HFD induced a strong vWAT expansion. However, and most interestingly, we found that in a subgroup of mice fed with the HFD (about 30%) the development of vWAT inflammation was limited. In particular, in this subgroup of HFD-fed mice, that we named Low-INFL mice, vWAT expression of *Ccl2, Cxcl12*, and *Itgax* and the circulating levels of insulin, resistin and leptin, which are correlated with vWAT inflammation, were significantly lower when compared to the other HFD-fed mice, there after named Hi-INFL (Fig. 1A, B). In contrast, the accumulation of vWAT was comparable in both Low-INFL and Hi-INFL groups, suggesting that, in Low-INFL mice, a healthier expansion of vWAT takes place in response to the HFD (Fig. 1C). We reasoned that this different susceptibility to the detrimental effects of HFD is of great interest to identify key molecular events participating to the fine-tuning of vWAT remodeling and expansion in obesity.

**Fig. 2:**
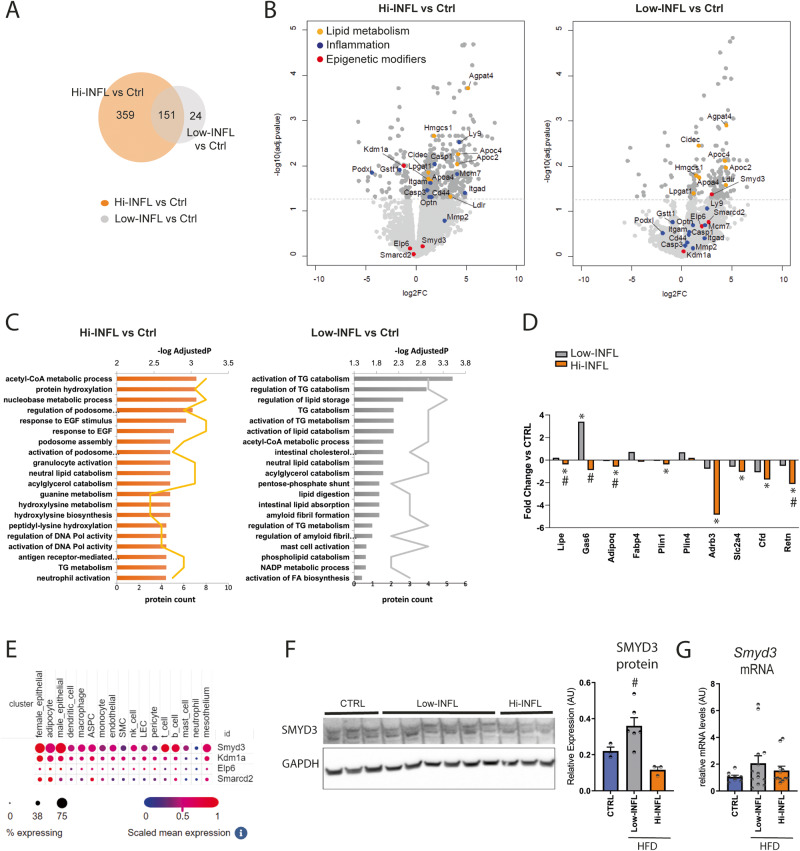
Proteomics analysis of vWAT of Low-INFL and Hi-INFL mice highlights SMYD3 as a possible player in their different response to the HFD. **A** Venn diagram showing the number of proteins whose levels are significantly changed by HFD in Hi-INFL (orange circle) and Low-INFL (grey circle), as calculated by Limma (adj.*p*-value < 0.05, FC > 1.5). *n* = 6 **B** Volcano plots corresponding to the comparisons Hi-INFL versus control group (left) and Low-INFL versus control group (right). Among the differentially expressed proteins (adj.*p*-value < 0.05), proteins involved in lipid metabolism are colored in yellow, proteins involved in inflammation are colored in blue, epigenetic modifiers are colored in red. **C** Biological pathways enriched within the differentially expressed proteins in Hi-INFL and Low-INFL with respect to control vWAT. **D** Fold changes of the expression of proteins involved in adipocyte function in Hi-INFL and Low-INFL as compared to control vWATs. * indicates significant changes versus control group; # indicates significant changes versus Low-INFL group, as calculated by Limma (adj.*p*-value < 0.05, FC > 1.5). Values are represented as mean and are in logarithmic scale. **E** mRNA expression of SMYD3, KDM1A, ELP6 and SMARCD2 in the single cell atlas of mouse adipose tissue [29]. ASPC adipocyte stem and progenitor cell precursors, SMC smooth muscle cells. LEC lymphatic endothelial cells. **F** Western Blot analysis of SMYD3 expression was performed in all Low-INFL vWATs analyzed in proteomics (*n* = 6) and three randomly picked control (*n* = 3) and Hi-INFL (*n* = 3) vWATs. Quantification of SMYD3 protein expression was performed for the lower band corresponding to 49 kDa. **G** mRNA levels of SMYD3 were measured in all residual mRNA samples from control (*n* = 8), Low-INFL (*n* = 13) and Hi-INFL (*n* = 12) vWATs. Bars represent mean ± SE. ^#^*P* < 0.05 vs. Hi-INFL group, as calculated by one-way ANOVA followed by Tukey’s multiple comparisons test.

To shed light on the global molecular pattern associated to the different response of Low-INFL and Hi-INFL mice, we performed a proteomic analysis of vWAT, which allowed the quantification of 6051 proteins. Among them, we found 175 and 510 differentially expressed proteins in Low-INFL and Hi-INFL, respectively, as compared to control vWATs (Fig. 2A). Volcano plots in Fig. 2B show the regulation profile of the 151 proteins commonly altered in both Low-INFL and Hi-INFL HFD groups. They include APOC2, APOC4, APOA4, LDLR, CIDEC, LPGAT1, AGPAT4, HMGCS1, whose function mainly relates to lipid metabolic processes, lipid transport, and endoplasmic reticulum stress (Fig. 2C and Supplementary Tables 2, 3). As expected, only the Hi-INFL vWAT proteome was enriched in proteins associated to inflammation (podosome regulation/activation, granulocyte and neutrophil activation, antigen receptor-mediated signaling pathway), such as MMP2, CASP1, CASP3, OPTN, ITGAM, ITGAD, LY9, PODXL, CD44, MCM7, GSTT1, reflecting the pro-inflammatory remodeling occurring within the tissue (Fig. 2B, C). This observation confirms at global scale that inflammation is mainly occurring in vWAT of Hi-INFL mice, as opposed to Low-INFL. To investigate which proteins and functions mainly differentiate the two HFD groups we performed supervised PLS-DA analysis [21]. As shown in Supplementary Fig. 1, while component 1 comprises the terms characterizing both Hi-INFL and Low-INFL compared to control, component 2, which accounts for about 6% of variability, includes the proteins discriminating Hi-INFL from Low-INFL vWATs. Of note, we found proteins involved in adipocyte function and marking adipocyte differentiation, such as adiponectin (ADIPOQ), hormone sensitive lipase (LIPE), fatty acid binding protein 4 (FABP4), resistin (RETN), and growth arrest-specific gene 6 (GAS6) as the five most discriminant variables of PLS-DA component 2 (Fig. 2D, Supplementary Table 4). The expression profile of some of these proteins was confirmed at mRNA level in all residual RNA samples (Supplementary Fig. 2). Overall, this expression pattern suggests that adipocyte differentiation is enhanced in Low-INFL compared to Hi-INFL, as further indicated by other proteins included in the dataset, such as the glucose transporter type 4 (SLC2A4), adipsin (CFD), beta-3 adrenergic receptor (ADRB3), perilipin 1 and 4 (PLIN1and PLIN4). Considering that all the mice used in this experiment shared the same genetic background and their genetic variability is very low, we hypothesized that epigenetic changes underlie the different behavior of Low-INFL and Hi-INFL groups. Therefore, we looked for chromatin modifier enzymes among the proteins that most differentiate Low-INFL from Hi-INFL vWAT, and we found four of them, including SET And MYND Domain Containing 3 (SMYD3), Elongator Acetyltransferase Complex Subunit 6 (ELP6), lysine (K)-specific demethylase 1 A (KDM1A) and SWI/SNF related, matrix associated, actin dependent regulator of chromatin, subfamily d, member 2 (SMARCD2) (Fig. 2B). Strikingly, GWAS studies have previously highlighted SNPs associated to phenotypes linked to inflammation [22] and/or obesity, such as BMI and waist to hip ratio in humans [23–26] for SMYD3, ELP6 and KDM1A. KDM1a (alias LSD1), whose expression is reduced only in Hi-INFL mice also at mRNA levels (Supplementary Fig. 2), was already described as a repressor of adipocyte inflammatory genes [27], which further validate our experimental setup. Conversely, no information was available about the possible role of SMYD3, ELP6 and SMARCD2 in adipose tissue. We therefore checked their expression in two comprehensive datasets of all cell types populating vWAT [28, 29]. SMYD3 was expressed in various adipose tissue cell types, including adipocytes, adipocyte progenitors and immune cells, in mouse, but also in human vWAT (Fig. 2E and Supplementary Fig. 3), while KDM1, ELP6 and SMARCD2 were expressed at very low levels. We thus focused our attention on SMYD3, a zinc binding protein with methyl-transferases activity, which gained attention in the last years as regulators of cell proliferation and developmental processes [30–32]. SMYD3 protein expression pattern highlighted in vWAT by proteomic analysis was confirmed by western blot analysis (Fig. 2F) and SMYD3 RNA levels showed a consistent expression pattern, although the changes did not reach statistical significance (Fig. 2G).

**Fig. 3:**
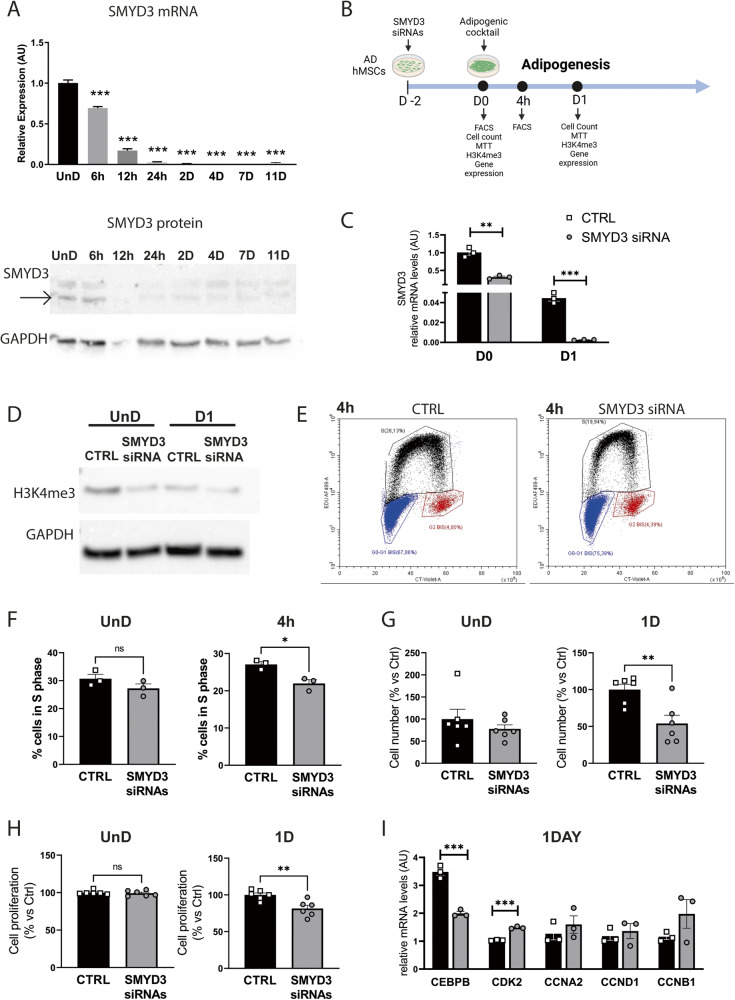
SMYD3 regulates cell proliferation at the beginning of adipocyte differentiation. **A** mRNA and protein levels of SMYD3 in Adipose-Derived hMSCs (AD-hMSCs), either undifferentiated (UnD) or differentiated for 6 h, 12 h, 24 h, 2 days, 4 days, 7 days and 11 days after the induction of adipocyte differentiation. *n* = 3. Bars represent mean ± SE. ^**^P < 0.01; ^***^P < 0.001 versus UnD samples, as calculated by one way ANOVA, followed by Dunnett’s multiple comparison test. **B** Experimental scheme (created with BioRender): siRNA of SMYD3 (siSMYD3) or with scrambled RNAs (CTRL) was performed in proliferating AD-hMSCs. 2 days after, adipocyte differentiation was induced. Cells were collected right before inducing differentiation (day 0), 4 h (4 h) or 24 h (day 1) after adipogenesis induction. **C** SMYD3 RNA levels at day 0 and day 1 (*n* = 3). **D** Representative image of the levels of H3K4me3 following SMYD3 siRNA at day 0 and day 1. GAPDH was used as loading control. **E** Representative image of FACS analysis of cell cycle 4 h after adipogenesis induction. Blue dots are cells in G0/G1 phase, black dots are cells in S phase, red dots are cells in G2 phase. **F** Percentage of cells in S phase at day 0 (UnD) and 4 h after adipogenesis induction following SMYD3 siRNA (*n* = 3). **G** Cell number (*n* = 6) and **H** cell proliferation by MTT assay (*n* = 6) were assessed at day 0 and day 1. One representative experiment is shown. **I** CEBPB, CDK2, CCNA2, CCND1 and CCNB1 mRNA levels were measured at day 1 (*n* = 3). Bars represent mean ± SE. ^**^P < 0.01; ^***^P < 0.001 versus CTRL samples, as calculated by student *t*-test. ns non significant.

We next explored the expression profile of SMYD3 in adipocytes by choosing a human in vitro model, namely Adipose-Derived human mesenchymal stem cells (AD-hMSCs) that can be differentiated into mature adipocytes [33]. Interestingly, we found that SMYD3 is expressed in differentiating AD-hMSCs, and its mRNA and protein levels decrease rapidly along the differentiation process (Fig. 3A).

**Fig. 4:**
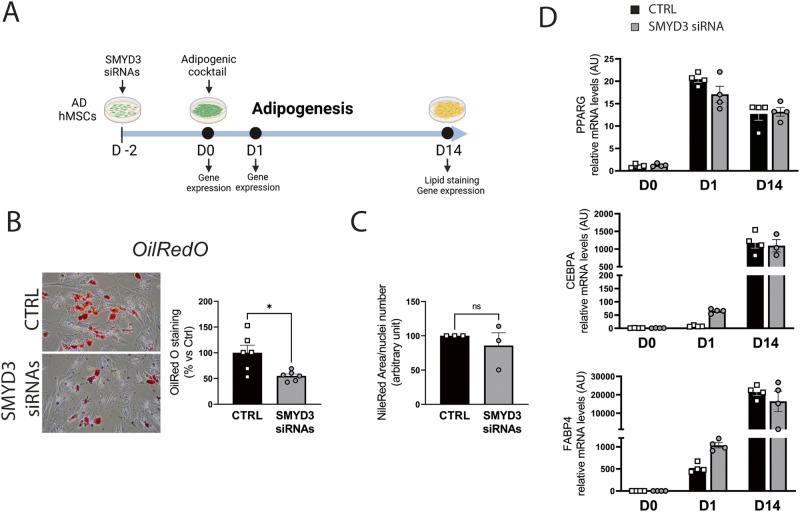
SMYD3 effects on lipid accumulation and adipogenesis. **A** Experimental scheme (created with BioRender): siRNA of SMYD3 (siSMYD3) or with scrambled RNAs (CTRL) was performed in proliferating Adipose-Derived hMSCs (AD-hMSCs). After two days, cells were differentiated to adipocytes and were collected 14 days after adipogenesis induction. **B** Oil Red O staining was used to stain neutral lipids in cells differentiated for 14 days (*n* = 6). Representative pictures of cells transfected with siRNAs of SMYD3 (siSMYD3) or with scrambled RNAs (CTRL) are shown and the graph reports Abs value of the extracted dye of one representative experiment. *P < 0.05 versus CTRL samples, as calculated by student *t*-test. **C** Graph representing the quantification of lipid content with Nile Red staining normalized for nuclei number at day 14 of differentiation (*n* = 3). *ns* = non significant, as calculated by non parametric Wilcoxon test. **D** mRNA levels of PPARG, CEBPA and FABP4 were measured at day 0, day 1 and day 14 (*n* = 4). Two-way ANOVA followed by followed by Sidak’s multiple comparison test (siSMYD3 vs. CTRL) was used. For the three genes, the time effect was significant, with *P* < 0.0001. Bars represent mean ± SE.

The high expression of SMYD3 in undifferentiated AD-hMSCs, prompted us to explore its role in the early steps of differentiation. We thus knocked down SMYD3 expression in proliferating AD-hMSCs two days before inducing adipocyte differentiation (day -2; Fig. 3B). We significantly blunted SMYD3 levels at the induction of adipogenesis (day 0) and the reduction was still significant in cells differentiated for one day (Fig. 3C and Supplementary Fig. 4). Given the known ability of SMYD3 to tri-methylate histone H3 at lysine 4 in other cell types [30], we first investigated whether it has this epigenetic activity also in differentiating AD-hMSCs. The levels of total H3K4me3 were strongly reduced by SMYD3 silencing both in undifferentiated (day 0) and one day differentiated cells (Fig. 3D), confirming the epigenetic role of SMYD3. We next checked whether SMYD3 knockdown had a direct effect on the inflammatory potential of differentiating AD-hMSCs, but no effect was observed on inflammatory markers such as IL1B, CCL2 and IL6 (Supplementary Fig. 5). SMYD3 is also known for its regulatory function of cell cycle progression. We thus assessed the consequences of SMYD3 silencing on cell proliferation by FACS analysis. While in undifferentiated cells EdU incorporation was similar in control and knocked-down cells, 4 h after the induction of adipogenesis the percentage of cells in S phase was significantly reduced by SMYD3 silencing (Fig. 3E, F). Accordingly, at day 0, no significant impact was observed on cell number and proliferation in undifferentiated AD-hMSCs (Fig. 3G, H). In contrast, when we checked the effect of SMYD3 silencing 24 h after the addition of the differentiation cocktail, we found that cell count and proliferation were significantly reduced in SMYD3 knocked-down AD-hMSCs (Fig. 3G, H). This is very interesting in light of the previous finding showing that several rounds of cell division occur also right after the induction of adipocyte differentiation in vitro, during the so-called mitotic clonal expansion (MCE), which is an important step for adipogenesis [34–36]. Our results suggest that SMYD3 might be involved in the regulation of cell proliferation during the MCE in AD-hMSCs at very early stages of adipocyte differentiation. To further explore this possibility, we checked the effects of SMYD3 knock-down on the expression of key regulators of MCE and cell proliferation, such as CCAAT/enhancer-binding protein beta (CEBPβ), Cyclin dependent kinase 2 (CDK2), and cyclins A2, B1 and D1 (CCNA2, CCNB1, CCND1). While no effects were observed on cyclins, SMYD3 knock-down was accompanied by a significant reduction of CEBPβ levels and increased levels of CDK2 (Fig. 3I). Of note, the effect of SMYD3 knock-down on cell proliferation at the beginning of adipocyte differentiation had also long-term consequences (Fig. 4A). Indeed, we found a reduced Oil Red O staining in SMYD3 silenced cells, as compared to control adipocytes (Fig. 4B), indicating a decreased total lipid accumulation. However, this difference was abolished when the lipid content was normalized by the number of cells (Fig. 4C and Supplementary Fig. 6), suggesting that the lack of SMYD3 reduces cell proliferation at the beginning of differentiation, but does not impair the adipogenesis of the remaining cells. Accordingly, we found that the expression of PPARG, CEBPA and FABP4 was not influenced by SMYD3 knock-down (Fig. 4D). Collectively, our results place SMYD3 as a new actor in the regulation of adipocyte precursor proliferation, in particular of the mitotic clonal expansion phase, possibly through modulation of CEBPβ and CDK2.

## Discussion

Obesity is characterized by an increase of adipose tissue mass, which is generally associated to a high predisposition toward metabolic diseases. Interestingly, some individuals with obesity seem protected from the detrimental metabolic consequences of obesity. Recent GWAS studies have identified several independent loci whose genetic variance is associated with the susceptibility to obesity-driven metabolic perturbations [37, 38]. However, these studies cannot explain the rapid increment observed in the rate of obesity worldwide [39–41], raising questions about the role of epigenetic mechanisms that are sensitive to environmental inputs and might contribute to regulate the individual vulnerability to obesity-driven detrimental consequences. Our study arises from an observation obtained in a large group of C57Bl6 mice fed with a high fat diet for 8 weeks, of which about 30% had a significantly lower vWAT inflammation and systemic insulin resistance, despite a similar gain in adipose tissue mass. These mice, that we called Low-INFL, seemed thus protected against the development of obesity-driven inflammation and related metabolic consequences observed in the other mice fed with HFD (Hi-INFL), representing an invaluable experimental group to shed light on new key determinants of the susceptibility to obesity-driven detrimental effects.

Further large-scale investigations of vWAT proteomes of these mice confirmed the enrichment of proteins associated to the onset of an inflammatory response specifically in Hi-INFL HFD mice. Most interestingly, we found that many proteins involved in adipogenesis and/or adipocyte differentiation, including ADIPOQ, PLIN1, PLIN4, LIPE etc. were differentially expressed in Hi-INFL, as compared to Low-INFL vWAT. Our finding is consistent with previous reports suggesting that adipogenesis, by favoring a healthier expansion of adipose tissue, would prevent the obesity-mediated metabolic decline [8, 42]. First, many genes associated with impaired expansion of adipose tissue are functionally associated with adipocytes/adipogenesis [37, 38, 43]. In line with these observations, WAT depots from patients with metabolic syndrome are enriched in hypertrophic adipocytes and proinflammatory macrophages and present hypoxia and fibrosis [44, 45]. Conversely, fat depots from metabolically healthy individuals contain a higher number of small adipocytes and have a relative high blood vessel density [46]. Several studies in mouse models also support the idea that the inability of WAT to adequately expand, to meet the energy storage demands, results in adipose tissue dysfunction. Pulse-chase genetic lineage tracing methods, which allow to track adipogenesis in vivo, have shown that a HFD rapidly triggers the commitment of adipocyte progenitors (APs), the first step necessary for adipogenesis [47]. However, anti-adipogenic signals appear upon prolonged HFD feeding, thus impairing the terminal differentiation of adipocytes [16]. Interestingly, such anti-adipogenic signals are activated preferentially in the visceral adipose tissue (vWAT) [16], which represents the fat depot more prone to develop obesity-related inflammation [48]. Further suggesting the tight link between adipogenesis rate and the onset of inflammation in obese WAT, selective stimulation of de novo adipocyte differentiation in Pdgfrβ+ preadipocytes was shown to protect against pathologic visceral adipose expansion and inflammation [49].

Our finding that in Low-INFL mice adipogenesis is enhanced compared to Hi-INFL mice after 8 weeks of HFD raise questions about the regulators of adipocyte differentiation underlying such difference, although we cannot exclude a role of inflammation in the blunted adipogenesis observed in the vWAT of Hi-INFL mice. Among the proteins showing a differential expression in Low-INFL and Hi-INFL vWAT, we found that KDM1a (alias LSD1) was significantly downregulated specifically in Hi-INFL mice. Such an expression profile, together with the known role of KDM1 as a promoter of adipogenesis [15], is consistent with a dampened adipogenesis in Hi-INFL vWAT. In addition, we found that the histone methyltransferase SMYD3 was significantly induced only in Low-INFL vWAT. The family of SMYD methyl-transferases (SET and MYND domain-containing proteins) are well known regulators of cancer cell proliferation [30–32]. More particularly, SMYD3 is frequently overexpressed in human cancers, and its high expression is associated with poor prognosis [50, 51]. Recently, SMYD3 was also implicated in physiological developmental processes, such as myogenesis [52, 53] and iTreg differentiation [54, 55], while its role in adipose tissue is unknown. Of note, mice lacking SMYD3 are viable and often their phenotype appears only upon a given challenge (i.e. tumor induction), which suggests a role of this protein in the fine-tuning of specific tissue/cell responses that might alter susceptibility to disease. Consistent with this idea, a differential DNA methylation pattern at the SMYD3 gene was recently found in insulin sensitive women with obesity [56]. Our results highlight for the first time SMYD3 as a new actor in the regulation of adipocyte physiology. SMYD3 expression declines rapidly with differentiation, suggesting that it plays a role early in the process of adipogenesis. Consistent with this hypothesis, SMYD3 seems involved in the regulation of the levels of H3K4 tri-methylation, confirming its epigenetic role [30] also in AD-hMSCs at the beginning of adipocyte differentiation. According to previous in vivo and in vitro findings, SMYD3 activity is critical for pathways regulating proliferation [31]. Cell proliferating activity was observed at the very beginning (first 60 h) of adipocyte differentiation, both in murine 3T3L1 and in AD-hMSCs [34–36, 57]. This process, referred to as mitotic clonal expansion (MCE), results in a three to four-fold increase of the total cell number and is a prerequisite for efficient adipogenesis in 3T3L1 cells [34]. Our data indicate that SMYD3 might be involved in the regulation of cell proliferation at the early stages of adipogenesis in AD-hMSCs. Indeed, its depletion prior adipogenesis induction reduces the number of cells, which, at longer term, might affect the total capacity for lipid storage, by decreasing the population of differentiating adipocytes, even without affecting the adipogenic process per se. Our observations are in line with previous findings in AD-hMSCs showing that, in human cells, the positive effect of cell proliferation stimulation on lipid accumulation mainly depends on the regulation of the number of cells available for the adipogenic process [36]. At the molecular levels, SMYD3 effect on preadipocyte proliferation might involve its impact on the expression of CEBPβ and/or CDK2 both participating in MCE. Interestingly, CDK2 was already identified as a SMYD3 target gene in hepatic cells [30]. Future studies will be necessary to fully unravel SMYD3 function in adipose tissue and to understand how its activity can be modulated in physiology and disease, more precisely in the context of healthier obesity.

### Supplementary information

Supplemental information

Supplementary Table 2

Supplementary Table 3

Supplementary Table 4

## Data Availability

The mass spectrometry proteomics data have been deposited to the ProteomeXchange Consortium via the PRIDE [58] partner repository with the dataset identifier PXD043165. All the other data and codes generated during and/or analyzed during the current study are available from the corresponding author on reasonable request.
