# Supplementary material for: SMYD3: a new regulator of adipocyte precursor proliferation at the early steps of differentiation
Source: Int J Obes (Lond). 2023 Dec 26;48(4):557–66. doi: 10.1038/s41366-023-01450-x (PMC10978492; doi:10.1038/s41366-023-01450-x)
Supplement: Supplementary file 1 — Supplemental information [file 41366_2023_1450_MOESM1_ESM.pdf]

SMYD3: a new regulator of adipocyte precursor proliferation at the early steps of differentiation

Tatjana Sajic[1,2], Chayenne Karine Ferreira Gomes[2], Marie Gasser[1,2], Tiziana Caputo[3], Nasim Bararpour[4,5], Esther Landaluce-Iturriria[6], Marc Augsburger[1], Nadia Walter[7], Alexandre Hainard[7], Isabel C. Lopez-Mejia[6], Tony Fracasso[8], Aurélien Thomas[1,2], Federica Gilardi[1,2]

1. Unit of Forensic Toxicology and Chemistry, CURML, Lausanne and Geneva University Hospitals, Lausanne, Geneva, Switzerland.
2. Faculty Unit of Toxicology, CURML, Faculty of Biology and Medicine, University of Lausanne, Lausanne, Switzerland.
3. Section on Integrative Physiology and Metabolism, Joslin Diabetes Center, Harvard Medical School, Boston, MA, USA
4. Stanford Center for Genomics and Personalized Medicine, Stanford, CA, USA
5. Department of Genetics, Stanford University, Stanford, CA, USA
6. Center for Integrative Genomics, University of Lausanne, Lausanne, Switzerland
7. Proteomics Core Facility, Faculty of Medicine, University of Geneva, Geneva, Switzerland
8. Unit of Forensic Medicine, CURML, Lausanne and Geneva University Hospitals, Lausanne, Geneva, Switzerland

Corresponding author:

Federica Gilardi PhD

Faculty Unit of Toxicology

Chemin de la Vulliette 4

1000 Lausanne 25

Switzerland

Phone number: +41 22 379 55 78

Supplementary Methods

Proteomics analysis

Proteomic analysis was performed starting from 30 mg of snap frozen visceral adipose tissue per mice. 6 individual samples were analyzed per experimental group (control, Low-INFL and Hi-INFL), for a total of 18 samples. Samples were homogenized in 500 µl of ice-cold phosphate-buffered saline (PBS, #10010015, Gibco) and tissue extracts were centrifuged for 5 min at 4°C at 3000 rpm. The soluble tissue proteomes in 400 µl of collected supernatant were purified by adding 100 µl of trichloroacetic acid (TCA) to a final 25% (v:v) concentration. After 1 h of protein TCA precipitations on ice, the protein pellets were collected at 16,000g for 15 min and washed three times with ice-cold acetone.

Purified protein pellets were dissolved in 8 M urea buffer, reduced at RT for 30 min with 5mM tris(2-carboxyethyl)phosphine hydrochloride (TCEP, #C4706, Sigma Aldrich) and alkylated in dark for 30 min with 10 mM of iodoacetamide (IAA, #I1149, Sigma Aldrich). Prior to digestion, protein pellets were diluted to 1 M urea concentration with 50mM Ammonium Bicarbonate and digested overnight with a ratio of 1 µg trypsin (#V5113, Promega) for 20 µg protein. Generated peptide digests were cleaned on MACROSpin Plate-Vydac Silica C18 (Nest Group Inc., Southborough, MA), solubilized in 30 µL of 0.1% aqueous formic acid (FA) with 2% acetonitrile (ACN). Indexed retention time (iRT) peptides were added (RT-kit WR, Biognosys) in equal 1 pmol/µL amount into each sample prior to MS injection.

Mass spectrometry analysis, raw data processing and protein quantification

The peptide digests of respective samples were processed by liquid chromatography-electrospray ionization tandem mass spectrometry (LC-ESI-MS/MS) on an Orbitrap Fusion Lumos Tribrid mass spectrometer (Thermo Fisher Scientific) equipped with an Easy nLC1200 liquid chromatography system (Thermo Fisher Scientific). Peptides were trapped on an Acclaim pepmap100, C18, 3µm,

75µm x 20mm nano trap-column (Thermo Fisher Scientific) and separated on a 75 µm x 500 mm, C18 ReproSil-Pur (Dr. Maisch GmBH), 1.9 µm, 100 Å, home-made column.

LC-ESI-MS/MS system was used for performing both data-dependent acquisition (DDA) and data-independent acquisition (DIA). Raw data search, generation of peptide and protein data matrices were performed with commercial proteomic software Spectronaut (version:14.8.201029.47784, Biognosys, https://biognosys.com/software/spectronaut/) as described previously (1, 2). In brief, 22 data files acquired in DDA mode were used for creation of tissue specific spectral library while DIA recorded 18 sample files were used for targeted data search and consistent peptide and protein quantification.

For DDA, the analytical separation was run for 180 min using a gradient of H2O/FA 99.9%/0.1% (solvent A) and CH3CN/H2O/FA 80.0%/19.9%/0.1% (solvent B). DDA was performed with MS1 full scan at a resolution of 120’000 FWHM followed by as many subsequent MS2 scans on selected precursors as possible within 3 second maximum cycle time. MS1 was performed in the Orbitrap with an AGC target of 4 x 105, a maximum injection time of 50 ms and a scan range from 400 to 1250 m/z. MS2 was performed in the Orbitrap at a resolution of 30’000 FWHM with an AGC target at 5 x 104 and a maximum injection time of 54 ms. Isolation windows was set at 1.6 m/z and 30% normalised collision energy was used for HCD. For creation of project-specific spectral library we used 22 raw DDA files recorded from both subcutaneous and visceral adipose tissue digests. The respective files were loaded in Spectronaut that also integrates Pulsar as search engine. We used default search parameters against the ex sp 10090.fasta mouse database (reviewed canonical Swiss-Prot mouse proteome database, released 2016.11.01) appended with common contaminants and reversed sequence decoys and iRT peptides sequence. Unique proteins identified at 1% of protein false discovery rate (FDR) were used to generate the list of proteins and their corresponding tryptic peptides included in spectral library.

For 18 DIA sample records, the analytical separation was run for 135 min using a gradient of H2O/FA 99.9%/0.1% (solvent A) and CH3CN/H2O/FA 80.0%/19.9%/0.1% (solvent B). DIA was performed with MS1 full scan at a resolution of 60,000 (FWHM) followed by 30 DIA MS2 scan with variable windows. MS1 was performed in the Orbitrap with an AGC target of 1 x 106, a maximum injection

time of 50 ms and a scan range from 400 to 1240 m/z. DIA MS2 was performed in the Orbitrap using higher-energy collisional dissociation (HCD) at 30%. Isolation windows was set to 28 m/z with an AGC target of 1 x 106 and a maximum injection time of 54 ms. Thus recorded DIA sample files and spectral library (.kit file extension) were loaded in Spectronaut DIA proteomics experiment for generation of quantitative data matrices. The respective proteins and peptide matrices at 1% FDR were generated by default settings (i.e. BGS factory settings) and exported from Spectronaut software as csv. Only tryptic peptides were used for protein quantification. To retain consistency of quantification, across the mouse cohort, we performed imputation of missing values for some non-detected protein quantities. Missing values were imputed optimally to avoid bimodal data distribution as described previously (3) by an in-house script sampling from a random distribution of values whose mean value corresponded to 75% of an empirically detected minimal precursor peptide value ± 0.5 of standard deviation (SD) obtained from respective measurements. Our analysis allowed the quantification of 6051 proteins at 1% protein and peptide FDR

Data Availability:

The mass spectrometry proteomics data have been deposited to the ProteomeXchange Consortium via the PRIDE (4) partner repository with the dataset identifier PXD043165.

Supplementary Figures

Supplementary figure 1

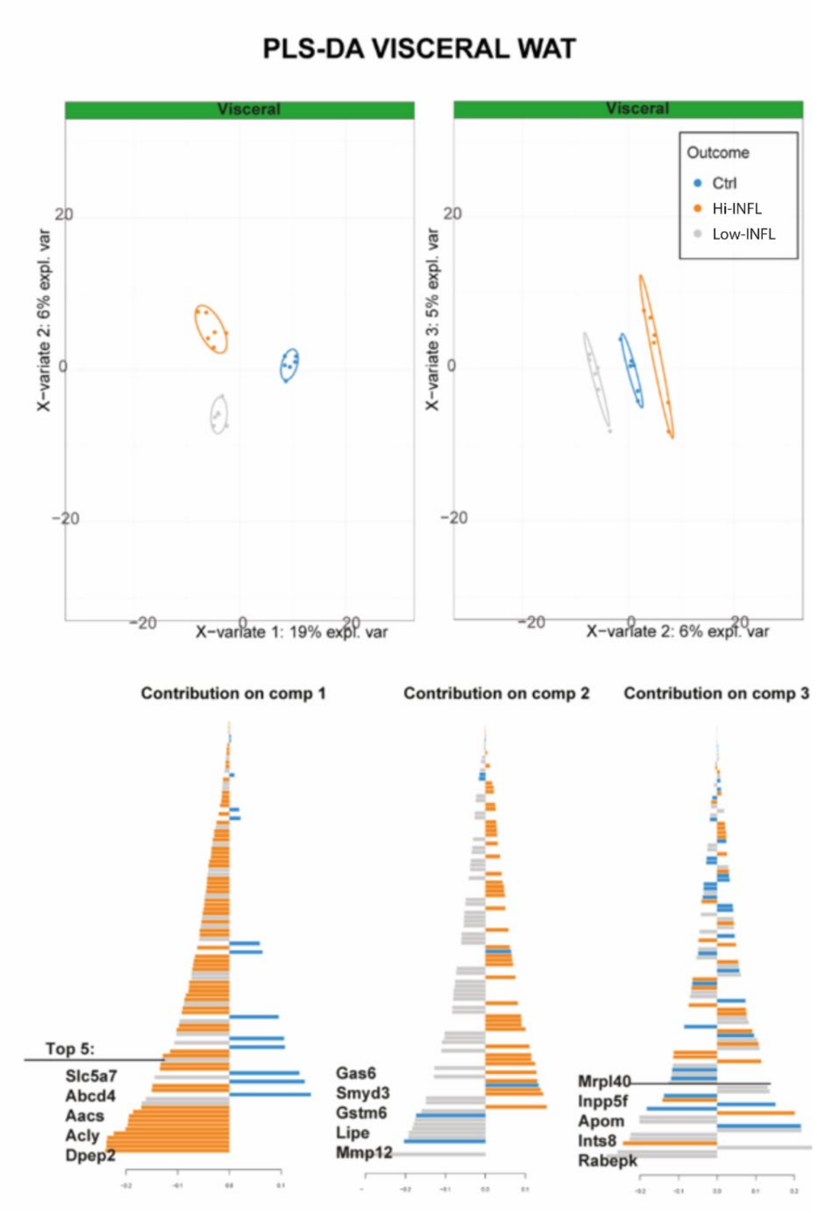

Partial Least-Squares Discriminant Analysis (PLS-DA) of protein levels in control, Low-INFL and Hi-INFL vWATs. We selected the 100 most descriptive proteins per each of three latent components correlating with the outcome of interest, namely control, Low-INFL and Hi-INFL. For each component the top 5 hits are indicated (the full list is provided in Supplementary Table 4).

Supplementary Figure 2

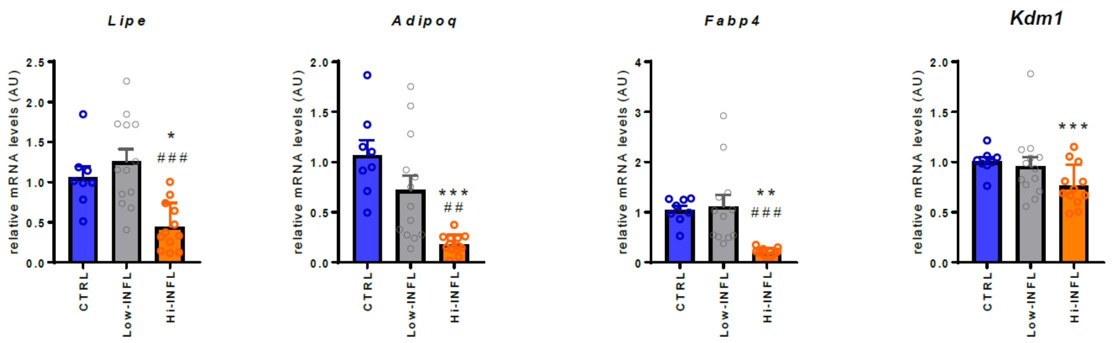

mRNA levels of Hormone sensitive lipase (*Lipe*), Adiponectin (*Adipoq*), Fatty acid binding protein 4 (*Fabp4*) and Lysine (K) specific demethylase 1A (*Kdm1*) were measured in all residual RNA samples from Control (CTRL), Low Inflammation (Low-INFL) and High Inflammation (High-INFL) vWAT. n=8 for control diet; n=13 for Low-INFL; n=12 for Hi-INFL. Bars represent mean ± SE. * P<0.05, ** P<0.01, *** P<0.001 versus control group; ## P<0.01, ### P<0.001 vs Low-INFL group, as calculated by one-way ANOVA followed by Tukey's multiple comparisons test.

Supplementary Figure 3

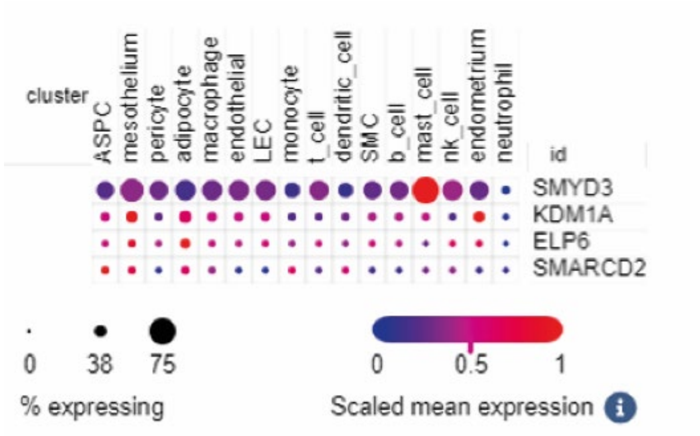

mRNA expression of SMYD3, KDM1A, ELP6 and SMARCD2 in the single cell atlas of human adipose tissue (5). ASPC: adipocyte stem and progenitor cell precursors; SMC: smooth muscle cells. LEC:lymphatic endothelial cells.

Supplementary Figure 4

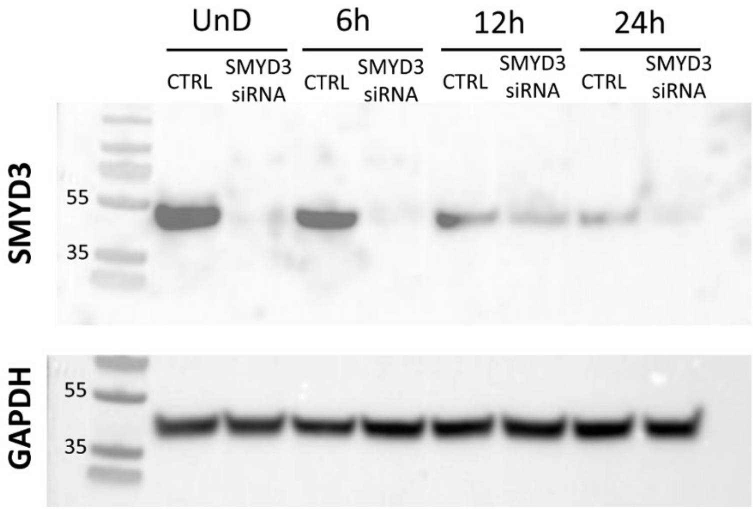

48h after SMYD3 silencing in AD-hMSCs, protein levels of SMYD3 were measured in undifferentiated cells (UnD) or 6h, 12h or 24h after the addition of the adipogenic cocktail, in control (CTRL) or knock-down cells (SMYD3 siRNA). SMYD3 band migrates at the expected molecular weight of 49kDa (6). GAPDH was used as loading control.

Supplementary Figure 5

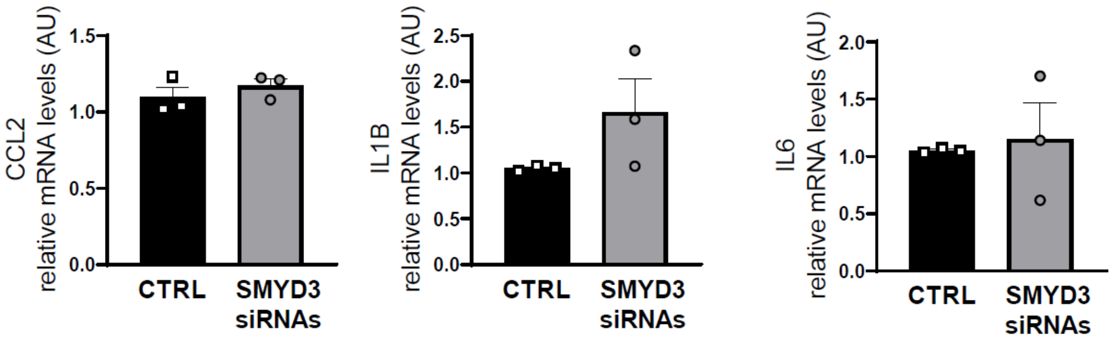

mRNA levels of CCL2, IL1B and IL6 were measured in AD-hMSCs, 48h after SMYD3 silencing and 24h after the addition of the adipogenic cocktail, in control (CTRL) and knock-down cells (SMYD3 siRNA). Student's T test was used for statistical analysis.

Supplementary Figure 6

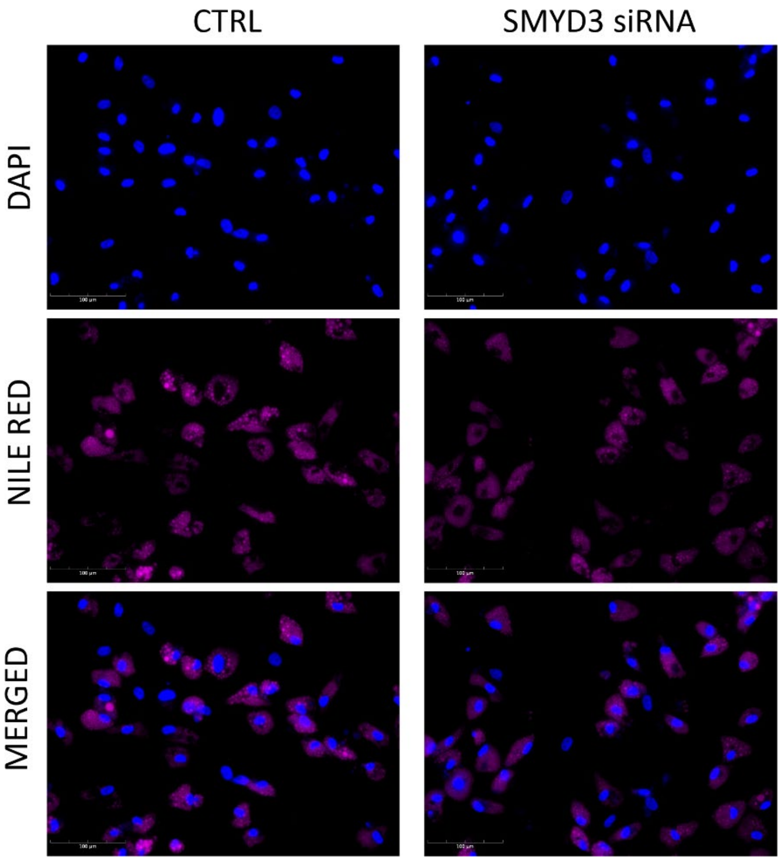

Representative pictures of nuclei (DAPI) and lipid staining (NILE RED) in AD-hMSCs differentiated to adipocytes for 14 days after transfection with siRNAs of SMYD3 (SMYD3 siRNA) or with scrambled RNAs (CTRL) two days before starting the differentiation. Scale bar = 100µm

Supplementary Tables

Supplementary Table 1

List of primers and probes used for Real-Time qPCR analysis of gene expression.

Primer name	species	primer (5'-3')
smyd3 FWD	mus musculus	TGACGAGCAAATATGGAAGGAG
smyd3 REV	mus musculus	GGTAGATGTTGATGTCGGGAAG
36b4 FWD	mus musculus	AGATGCAGCAGATCCGCAT
36b4 REV	mus musculus	GTTCTTGCCCATCAGCACC
itgax (cd11c)- FWD	mus musculus	TTCAAGGAGACAAAGACCCG
itgax (cd11c)- REV	mus musculus	AGAGAAAAGTTGAGGCGAAGAG
ccl2 FWD	mus musculus	GTCCCTGTCATGCTTCTGG
ccl2 REV	mus musculus	GCTCTCCAGCCTACTCATTG
cxcl12 FWD	mus musculus	AGTGAACTGCGCTGTCAATG
cxcl12 REV	mus musculus	GCCCTTGAGAGTGGCTATGA
adipoq FWD	mus musculus	GGAGATGCAGGTCTTCTTGG
adipoq REV	mus musculus	GCCCTTCAGCTCCTGTCAT
adipoq probe	mus musculus	CCTAAGGGTGAGACAGGAGATGTTGGA
lsd1 FWD	mus musculus	GTG TTC TGG GAC CCA AGT GT
lsd1 REV	mus musculus	TAA TGC CAG CAG CTT CTC CT
lipe FWD	mus musculus	GCTCCCTTTCCCCGA
lipe REV	mus musculus	ATGCAGAGATTCCCACCT
lipe probe	mus musculus	CACTGTGACCTGCTTGGTTCAACT
fabp4 FWD	mus musculus	GAC GAC AGG AAG GTG AAG AG
fabp4 REV	mus musculus	ACA TTC CAC CAC CAG CTT GT
RPS13 FWD	homo sapiens	CGTCCCCACTTGGTTGAAG
RPS13 REV	homo sapiens	TTGTGCAACACCATGTGAATC
RPS13 probe	homo sapiens	TGACATCTGACGACGTGAAGGAGCA
SMYD3 FWD	homo sapiens	CGTGTATTGGCTTTTCTGGG
SMYD3 REV	homo sapiens	CTGGGTTTGCAGCTTTTAAGG
SMYD3 probe	homo sapiens	ACACTTAGCACTACAGTATTTGGCGACG
PPARG FWD	homo sapiens	TCTCATAATGCCATCAGGTTTG
PPARG REV	homo sapiens	ATCTCCGCCAACAGCTTCT
PPARG probe	homo sapiens	CGGATGCCACAGGCCGAGAA
CEBPA FWD	homo sapiens	GCAAACTCACCGCTCCAATG
CEBPA REV	homo sapiens	GGAAGGAGGCAGGAAACCTC
CEBPB FWD	homo sapiens	TTTGTCCAAACCAACCGCAC
CEBPB REV	homo sapiens	GCATCAACTTCGAAACCGGC
CDK2 FWD	homo sapiens	TTTTGGAGTCCCTGTTCGTAC
CDK2 REV	homo sapiens	CGAGTCACCATCTCAGCAAAG
CCNA2 FWD	homo sapiens	CTGCATTTGGCTGTGAACTAC
CCNA2 REV	homo sapiens	ACAAACTCTGCTACTTCTGGG

CCND1 FWD	homo sapiens	CATCTACACCGACAACTCCATC
CCND1 REV	homo sapiens	TCTGGCATTTTGGAGAGGAAG
CCNB1 FWD	homo sapiens	GGCTTTCTCTGATGTAATTCTTGC
CCNB1 REV	homo sapiens	GTATTTTGGTCTGACTGCTTGC
FABP4 FWD	homo sapiens	GAAAGAAGTAGGAGTGGGCTT
FABP4 REV	homo sapiens	GGCCCAGTATGAAGGAAATCT
FABP4 probe	homo sapiens	CTGGCATGGCCAAACCTAACATGA
CCL2 FWD	homo sapiens	AGCAAGTGTCCCAAAGAAGC
CCL2 REV	homo sapiens	TGAACCCACTTCTGCTTGG
CCL2 probe	homo sapiens	ATCTTCAAGACCATTGTGGCCAAGG
IL1B FWD	homo sapiens	ATCTTCATTGCTCAAGTGTCTGAA
IL1B REV	homo sapiens	CTGGAAGGAGCACTTCATCTG
IL1B probe	homo sapiens	CAGAAGTACCTGAGCTCGCCAGTGAAA
IL6 FWD	homo sapiens	CAATGAGGAGACTTGCCTGGT
IL6 REV	homo sapiens	CAGGAACTGGATCAGGACTTTT
IL6 probe	homo sapiens	AATCATCACTGGTCTTTGGAGTTTGAGGT

Supplementary Table 2

Fold change and LIMMA statistical analysis of the expression of the 6051 proteins that were confidently quantified by proteomics analysis in vWAT of Hi-INFL vs CTRL and Low-INFL vs CTRL mice. n=6 individual samples/experimental group.

Supplementary Table 3

List of the Gene Ontology (GO) pathways enriched in differentially expressed proteins in vWAT of Hi-INFL vs CTRL and Low-INFL vs CTRL mice.

Supplementary Table 4

List of the 100 most descriptive proteins of the three latent components correlating with the outcome of interest, namely CTRL, Low-INFL and Hi-INFL vWATs, as determined by Partial Least-Squares Discriminant Analysis (PLS-DA).

References

1. Muntel J, Gandhi T, Verbeke L, Bernhardt OM, Treiber T, Bruderer R, et al. Surpassing 10 000 identified and quantified proteins in a single run by optimizing current LC-MS instrumentation and data analysis strategy. Mol Omics. 2019;15(5):348-60.
2. Amon S, Meier-Abt F, Gillet LC, Dimitrieva S, Theocharides APA, Manz MG, et al. Sensitive Quantitative Proteomics of Human Hematopoietic Stem and Progenitor Cells by Data-independent Acquisition Mass Spectrometry. Mol Cell Proteomics. 2019;18(7):1454-67.
3. Tyanova S, Temu T, Sinitcyn P, Carlson A, Hein MY, Geiger T, et al. The Perseus computational platform for comprehensive analysis of (prote)omics data. Nat Methods. 2016;13(9):731-40.
4. Perez-Riverol Y, Bai J, Bandla C, Garcia-Seisdedos D, Hewapathirana S, Kamatchinathan S, et al. The PRIDE database resources in 2022: a hub for mass spectrometry-based proteomics evidences. Nucleic Acids Res. 2022;50(D1):D543-D52.
5. Emont MP, Jacobs C, Essene AL, Pant D, Tenen D, Colleluori G, et al. A single-cell atlas of human and mouse white adipose tissue. Nature. 2022;603(7903):926-33.
6. Codato R, Perichon M, Divol A, Fung E, Sotiropoulos A, Bigot A, et al. The SMYD3 methyltransferase promotes myogenesis by activating the myogenin regulatory network. Sci Rep. 2019;9(1):17298.
